# A novel oncolytic herpes simplex virus type 2 induces polarization of tumor-associated macrophages and remodels the immune microenvironment in glioblastoma

**DOI:** 10.1007/s00262-026-04440-w

**Published:** 2026-05-30

**Authors:** Xin Yang, Shenglan Li, Yanjie Lan, Can Wang, Meng Zhang, Wenjing Zhao, Rong Zhang, Mengqian Huang, Zhuang Kang, Botao Zhang, Xiangyong Gu, Feng Chen, Zhen Wu, Binlei Liu, Wenbin Li

**Affiliations:** 1 Department of Neuro-Oncology, Cancer Center, Beijing Tiantan Hospital, Capital Medical University, Beijing, China; 2 Department of Hematology, The Second Hospital of Tianjin Medical University, Tianjin, China; 3 Hepato-Pancreato-Biliary Center, School of Clinical Medicine, Beijing Tsinghua Changgung Hospital, Tsinghua University, Beijing, China; 4 Wuhan Binhui Biopharmaceutical Co. Ltd., Wuhan, China; 5 Department of Neurosurgery, Beijing Tiantan Hospital, Capital Medical University, Beijing, China; 6 National “111” Center for Cellular Regulation and Molecular Pharmaceutics, Key Laboratory of Fermentation Engineering (Ministry of Education), Hubei Provincial Cooperative Innovation Center of Industrial Fermentation, College of Bioengineering, Hubei University of Technology, Wuhan, China

**Keywords:** Glioblastoma multiforme, Oncolytic herpes simplex virus type 2 (OH2), Tumor immune microenvironment, JAK-STAT1 signaling, Immunotherapy

## Abstract

**Background:**

Glioblastoma multiforme (GBM) is a highly aggressive primary malignant brain tumor with limited survival despite multimodal therapy. Its profoundly immunosuppressive tumor microenvironment severely restricts treatment efficacy. Oncolytic viruses represent a promising therapeutic strategy, yet the immunological mechanisms underlying the antitumor activity of OH2 (oncolytic herpes simplex virus type 2) remain poorly defined.

**Methods:**

The effects of OH2 were evaluated in GBM cell lines (U251, LN229 and GL261) by assessing cell viability, migration, apoptosis, and cell cycle distribution. Modulation of tumor-associated macrophages (TAMs) was examined in RAW264.7 and THP-1 cells through functional assays, RNA sequencing, Western blotting and pharmacologic inhibition. In vivo studies were performed in subcutaneous and orthotopic GBM models to assess tumor growth and immune alterations. Exploratory clinical observations were conducted in seven recurrent GBM patients (NCT05235074), including peripheral blood monocyte profiling, longitudinal immune monitoring and cytokine analysis.

**Results:**

OH2 induced apoptotic and oncolytic cell death in GBM cells, while inhibiting cell proliferation and migration in vitro. In macrophage models, exposure to OH2-conditioned media enhanced proliferation and phagocytic activity and promoted M1-like polarization, accompanied by activation of JAK-STAT1 signaling. In vivo, OH2 suppressed tumor growth, promoted M1-like TAM polarization, reduced immunosuppressive macrophage populations, and increased recruitment of bone marrow-derived macrophages into the brain. Exploratory clinical observation suggested that changes in peripheral monocyte profiles may be associated with clinical benefit.

**Conclusions:**

OH2 exerts antitumor activity in GBM through direct oncolysis and immune microenvironment modulation, highlighting its potential as an immunotherapeutic strategy.

**Supplementary Information:**

The online version contains supplementary material available at 10.1007/s00262-026-04440-w.

## Background

Glioblastoma multiforme (GBM) is the most common and aggressive primary malignant brain tumor in adults, characterized by rapid proliferation, invasiveness, and resistance to conventional therapies [1, 2]. Despite advances in surgery, radiotherapy, and chemotherapy, prognosis remains poor, with a median overall survival of approximately 15 months and a 5-year survival rate below 5% [3, 4]. The addition of tumor-treating fields (TTFields) to temozolomide can extend median survival to nearly 21 months; however, recurrence is almost inevitable, underscoring the urgent need for innovative therapeutic strategies [5].

Oncolytic viruses (OVs) have emerged as promising therapeutic agents due to their ability to selectively replicate in tumor cells and elicit systemic antitumor immunity [6, 7]. Among them, oncolytic herpes simplex viruses (oHSV) are particularly attractive for GBM owing to their neurotropism, high replication efficiency, and ability to modulate the immunosuppressive tumor microenvironment (TME) [8–10]. Preclinical studies have shown that oHSV can directly lyse glioma cells and promote immune-mediated clearance [11–13]. However, to date, only one oHSV, teserpaturev/G47Δ (Delytact®), has been approved for GBM, highlighting the need for additional mechanistic and translational research [14–16].

A major barrier to GBM therapy is its profoundly immunosuppressive TME, where tumor-associated macrophages (TAMs), including infiltrating macrophages and resident macrophages, constitute the predominant immune cell population [17, 18]. TAMs are mostly polarized toward the M2-like pro-tumor phenotype, supporting tumor growth, angiogenesis, and immune evasion through anti-inflammatory cytokines such as TGF-β and IL-10 [18–20]. In contrast, M1-like TAMs exert pro-inflammatory and antitumor functions. Consequently, reprogramming TAMs from an M2-like phenotype to an M1-like phenotype is considered a promising strategy to enhance antitumor immunity in GBM [21–23].

Oncolytic herpes simplex virus type 2 (OH2) is a novel OV engineered to delete ICP34.5 to reduce neurotoxicity and enhance tumor selectivity, delete ICP47 to improve oncolytic activity, and express granulocyte–macrophage colony-stimulating factor (GM-CSF) to stimulate systemic antitumor immunity [24–26]. Preclinical studies have shown that OH2 monotherapy can eliminate tumors, induce tumor-specific immunity, and generate immune memory in colorectal cancer models [27, 28], while combination therapy promotes M1-like macrophage polarization and enhances antitumor responses [29]. Our preliminary observations in GBM models suggest that OH2 inhibits tumor proliferation and is associated with broader alterations in the immune landscape [30]. However, how OH2 regulates TAM polarization within the GBM microenvironment and how these changes relate to brain-resident and systemic immune responses remain insufficiently defined, limiting its translational advancement in glioblastoma.

In this study, we evaluated the antitumor efficacy and immunomodulatory effects of OH2 in GBM. We assessed its direct cytotoxic effects on GBM cells in vitro, validated antitumor activity in subcutaneous and orthotopic mouse models, investigated TAM polarization and associated signaling pathways, and characterized both brain and peripheral immune response, including peripheral monocytes and cytokine profiles. These findings provide mechanistic insights into OH2-mediated antitumor activity in GBM and support its further investigation in translational and clinical settings.

## Materials and methods

### Cell culture

U251 (RRID: CVCL_0021), LN229 (RRID: CVCL_0393), GL261 (RRID: CVCL_Y003), and RAW264.7 (RRID: CVCL_0493) cells were cultured in DMEM (Gibco, China), and THP-1 (RRID: CVCL_0006) cells were maintained in RPMI-1640 (Gibco, China). LN229 cultures were supplemented with 5% FBS (Gibco, Australia), whereas other cell lines were supplemented with 10% FBS. All media contained 1% penicillin–streptomycin (P/S, Biosharp, China). Cells were maintained at 37 °C with 5% CO₂.

### Conditioned medium (CM)

Conditioned medium (CM) was generated from U251 or GL261 cells treated with OH2 at different multiplicities of infection (MOI). Cells were seeded in 10-cm culture dishes at 70–80% confluence, washed with PBS, and incubated with 5 mL serum-free DMEM with or without OH2 (MOI = 0, 0.5, 1, 2) for 1h. An additional 5 mL DMEM containing 10% FBS was then added, and cells were incubated for 48h. Supernatants were collected, centrifuged at 500 g for 15 min at 4 °C, and passed through a 0.22 µm filter to remove cellular debris. Subsequently, the supernatants were subjected to UV inactivation for 40 min on ice to inactivate residual infectious virus. The effectiveness of viral inactivation was verified by the absence of cytopathic effects (CPE) in susceptible GBM cells (U251 and GL261). The clarified CM was stored at -80 °C for subsequent experiments.

### Cell viability assay

U251 and LN229 cells (3 × 10^3^ cells/well) were seeded in 96-well plates with three parallel wells and treated with OH2 at MOI = 0, 0.1, 0.5, 1, 2 or 4 for 0, 24, 48, or 72h. GL261 cells were cultured under the same conditions for 0, 12, 24, 36, or 48h. RAW264.7 cells (3 × 10^3^ cells/well) were allowed to adhere overnight and then treated with CM prepared under different OH2 MOIs (0, 0.1, 0.5, 1, 2) for 0, 12, 24, 36, or 48h. Cell viability was measured using the Cell Counting Kit-8 (CCK-8; Biosharp, China).

### 3D cell viability assay

U251 and GL261 cells (3 × 10^3^ cells/well) were seeded in 96-well low attachment plates in three parallel wells. After incubation for 72h, tumor spheroid formation was observed and treated with OH2 at MOI = 0, 1 for 6 days. The viability of the spheroids was assessed by luminescent signal using the CellTiter-Glo 3D Cell Viability Kit (Promega, USA).

### Cell apoptosis assay

U251 and GL261 cells (5 × 10^5^ cells/well) were seeded in 6-well plates in three parallel wells and allowed to adhere overnight. Cells were then treated with OH2 (MOI = 0, 0.5, 1, 2) for 48h, collected, and stained with Annexin V-FITC/PI (Apoptosis Kit, MultiScience, China). After staining for 5 min at room temperature, apoptotic cells were analyzed by flow cytometry (FCM) (Beckman, USA).

### Cell cycle assay

U251 cells were treated with OH2 (MOI = 0, 0.5, 1, 2) for 48h, harvested, resuspended, and fixed in pre-cooled 70% ethanol at − 20 °C overnight. Cells were then stained with propidium iodide (PI) solution (Beyotime, China) for 15 min at room temperature. Cell cycle distribution was analyzed by FCM.

### Wound healing assay

U251 and GL261 cells (1 × 10^6^ cells/well) were seeded in 6-well plates and cultured to approximately 90% confluence. Linear scratches were generated using a sterile 200 µL pipette tip, and detached cells were removed by washing with PBS. Cells were then incubated in serum-free DMEM supplemented with 1% P/S and treated with OH2 (MOI = 0, 0.5, 1, 2) for 48h. Wound closure was quantified as the percentage change in the initial scratch width.

### Transwell migration assay

U251 and GL261 cells (5 × 10^4^ cells/well) were seeded in Transwell inserts (8 μm, Corning, USA) with 500 μL serum-free culture medium containing OH2 (MOI = 0.5,1,2) or without OH2 (MOI = 0). The lower chamber was filled with 600 μL culture medium containing 20% FBS. After incubation for 24h, the Transwell inserts were fixed with 4% paraformaldehyde and stained with 0.1% crystal violet solution. Cells were imaged under a microscope.

### Polarization of RAW264.7 and THP-1 cell lines

RAW264.7 (1 × 10^6^ cells/well) cells were seeded into 6-well plates, followed by treatment with GL261-derived CM. After 48h, cells were harvested and stained with the antibodies against CD45, CD11b, F4/80, CD86, and CD206 for FCM analysis. THP-1 cells were differentiated into macrophage-like cells (M0-state) by treatment with 100 ng/ml phorbol 12-myristate 13-acetate (PMA, MCE, USA) for 48h, followed by a 24-h resting period. Cells were then treated with U251-derived CM for 48h, stained with antibodies against CD68, CD86, CD206, and analyzed by FCM. Antibodies details are provided in Supplementary Table 1.

### Enzyme-linked immunosorbent assay (ELISA)

RAW264.7 cells (2 × 10^5^ cells/well) were seeded into 12-well plates and treated with GL261-derived CM for 48h. THP-1 cells (M0-state) were treated with U251-derived CM under the same conditions. Cytokine concentrations in clarified culture supernatants and cytokines in CM were quantified using ELISA kits (MultiSciences, China). Absorbance was measured at 450 nm and cytokine concentrations were calculated using standard curves.

### Western blotting (WB)

RAW264.7 and THP-1 (M0-state) cells were treated with CM for 48h, harvested, and lysed in RIPA buffer (Solabio, China) on ice. Protein concentrations were determined using the BCA assay. Equal amounts of protein were separated by SDS-PAGE (NCM Biotech, China) and transferred to PVDF membranes (Millipore, USA). Membranes were blocked with 5% non-fat milk and incubated with primary antibodies overnight at 4 °C, followed by incubation with HRP-conjugated secondary antibodies for 2h at room temperature. Protein bands were visualized using an enhanced chemiluminescence (ECL) reagent (Bio-Rad, USA). Antibody details are shown in Supplementary Table 2.

### Phagocytosis assay

GL261 cells were labeled with CFSE using the CFSE Cell Division Tracker Kit (BioLegend, China) according to the manufacturer’s protocol. The CFSE-labeled GL261 cells were resuspended in complete DMEM at 2 × 10^6^ cells/mL. RAW264.7 cells cultured with CM were co-incubated with CFSE-labeled GL261 cells at a 1:5 ratio (tumor cells: macrophages) at 37 °C for 6h. Phagocytosis was evaluated by FCM, and the percentage of CFSE^+^F4/80^+^ cells was quantified.

### RNA sequencing (RNA-seq)

RAW264.7 and THP-1 (M0-state) cells were treated with CM prepared under different OH2 MOIs (0 or 1) for 48h. Total RNA was extracted using TRIzol reagent (Thermo Fisher Scientific, USA). Three biological replicates per group were prepared for both cell lines and submitted to Novogene (Beijing, China) for sequencing. Differentially expressed genes (DEGs) were identified using R software.

### Real-time quantitative PCR (RT-qPCR)

Total RNA was extracted using TRIzol reagent and cDNA was synthesized using a reverse transcription kit (TransGen, China). qPCR was performed using SYBR Green Master Mix (Thermo Fisher Scientific, USA) on an Applied Biosystem real-time PCR system. GAPDH was used as the internal reference. Relative gene expression levels were calculated using the 2^−ΔΔCt^ method. Primer sequences are listed in Supplementary Table 3.

### Inhibitor assays

To assess whether JAK-STAT1 activity is required for OH2-associated macrophage polarization, RAW264.7 cells and THP-1 (M0-state) cells were treated with CM in the presence of JAK1/2 inhibitor ruxolitinib (5 μM, MCE, USA) for 48h. CM derived from tumor cells not exposed to OH2 (MOI = 0) was used as the control condition under JAK inhibition.

### Animals and animal housing

Healthy female athymic nude mice (4–6 weeks old) and C57BL/6 mice (6 weeks old) were purchased from SPF Biotechnology Company (Beijing, China). Mice were housed under specific pathogen-free conditions at the Beijing Neurosurgical Institute. All animal experiments were performed in accordance with institutional guidelines and approved ethical protocols.

### Subcutaneous tumor xenograft models

U251 cells (5 × 10^6^ per mouse) were suspended in 100 μL PBS and injected subcutaneously into the right axilla of nude mice (n = 6 per group). Tumor formation was monitored, and when tumors reached 3–5 mm in diameter, mice were randomly assigned to receive intratumoral injections of OH2 (1 × 10^5^ or 1 × 10⁶ CCID₅₀) or PBS as control. Treatments (50 μL per injection) were administered on days 10, 14, and 18 after tumor cell inoculation. Tumor size was measured every 3 days using Vernier calipers, and tumor volume was calculated as V = a × b^2^/2, where a represents tumor length and b represents tumor width. Body weight was recorded every 3 days. On day 28, mice were euthanized, and tumors were excised, weighed and measured. Tumor tissues were processed for immunohistochemistry (IHC) and digested into single-cell suspensions for FCM, as previously described [31, 32].

### Intracranial tumor xenograft models

GL261 cells (4 × 10^4^ per mouse) suspended in 3 μL PBS were stereotactically implanted into the right striatum of 6-week-old C57BL/6 mice under isoflurane anesthesia. Injections were performed at coordinates 2 mm lateral to bregma, 1 mm anterior to the coronal suture, and 3 mm below the skull surface at a rate of 0.6 μL/min. Mice were randomly assigned to the OH2 and PBS control groups (n = 5 per group), and intratumoral injections of OH2 (1 × 10^7^ CCID_50_) or PBS (3 μL) were administered on days 8, 11, and 14 post tumor cell inoculation. Tumor progression was monitored by MRI (Bruker, 7.0 T) on days 7 and 20. Body weight and survival were monitored daily. Mice were euthanized on day 21 or earlier if they exhibited signs of distress, ataxia, or > 10% body weight loss. Brains were collected for multiplex immunofluorescence staining (mIF), and peritumoral tissues were processed into single-cell suspensions for FCM analysis. Peripheral blood was collected weekly for immune profiling, and a separate cohort of mice was followed for survival analysis.

### Hematoxylin–eosin (H&E) staining and IHC

Subcutaneous and intracranial tumor tissues were fixed in 4% paraformaldehyde, embedded in paraffin, and sectioned at 5 μm thickness. Sections were dewaxed, rehydrated and subjected to H&E staining using standard protocols. For IHC, paraffin-embedded sections were incubated with the primary antibodies and secondary antibodies, and developed with DAB substrate. Images were acquired using an optical microscope (Zeiss, Germany). Antibody details are shown in Supplementary Table 4.

### Multiplex immunofluorescence staining (mIF)

mIF was performed on paraffin-embedded brain sections using a 4-color IHC kit (Absin, Cat#50028). Sections were sequentially incubated with primary antibodies against F4/80, iNOS and CD206, followed by HRP-conjugated secondary antibodies and tyramide signal amplification. Nuclei were counterstained with DAPI. Multispectral images were acquired using the PhenoImager HT system (Akoya Biosciences, USA). Antibody details are shown in Supplementary Table 4.

### FCM analysis of tumor, brain, and peripheral blood

Tumor tissues were mechanically dissociated and enzymatically digested into single-cell suspensions using DNase I (Roche, Cat#10104159001) and collagenase type IV (Gibco-Life Technologies, Cat#17104-019) [32]. Red blood cells were lysed and cells were stained with fluorochrome-conjugated antibodies against CD45, CD11b, F4/80, CD86, and CD206, along with corresponding isotype controls. Single-cell suspensions from brain tissues were prepared as described previously [31, 33] and stained with antibodies against CD45, CD11b, F4/80, Ly6G, Ly6C, CD3, CD19, and L/D. For peripheral blood analysis, 20 μL of whole blood was collected from the caudal vein and incubated with antibodies against CD45, CD11b, CD115, Ly6G, Ly6C, CD3, and CD19 for 20 min on ice, followed by red blood cell lysis. Data were acquired on a Beckman Coulter flow cytometer and analyzed using Flowjo software. Antibody details are shown in Supplementary Table 1.

### Clinical samples analyses

Clinical samples were obtained from seven patients with recurrent GBM enrolled in an ongoing phase I/II clinical trial of OH2 (NCT05235074) at Beijing Tiantan Hospital, Capital Medical University (ethics approval: YW2021-031). All procedures followed the Declaration of Helsinki and ICH-GCP guidelines, and written informed consent was obtained. Patients received OH2 (1 × 10^7^ CCID_50_/1 mL) via intracapsular injection through an Ommaya reservoir every 21 days.

Among the 7 enrolled patients after one treatment, those with progressive disease (PD) were assigned to the clinical non‑benefit group, while those with stable disease (SD), partial response (PR) or complete response (CR) were assigned to the clinical benefit group. Statistical analyses of monocytes were performed on routine blood test results obtained before Cycle 1 treatment and at the time of response assessment after Cycle 1 treatment.

Peripheral blood mononuclear cells (PBMCs) were collected at baseline and multiple treatment cycles and were isolated by using lymphocyte separation medium (Stemcell, Canada). Cells were stained with CD45-FITC, CD14-APC, and CD16-PE for flow cytometry, the details of antibodies are provided in Supplementary Table 1. Longitudinal changes in peripheral CD14⁺CD16^−^ monocyte subsets were modeled by simple linear regression and the regression slope was used to quantify immune dynamics.

Plasma and cerebrospinal fluid (CSF) were collected at baseline and multiple treatment cycles. Cytokine concentrations were quantified by cytometric bead arrays (RayBio, Cat#FAH-STRM-1-96). Exploratory cytokine profiling between PFS groups were performed using limma package (R v4.x). Cytokine patterns were visualized by volcano plots (ggplot2).

### Statistical analysis

Statistical analyses were performed using GraphPad Prism version 10 and R (v4.x). Data are presented as mean ± standard deviation (SD) unless otherwise indicated. Unpaired Student’s t-test was used for comparisons between two groups, and one-way ANOVA was applied for comparisons among multiple groups. Kaplan–Meier survival curves were compared using the log-rank test. Correlations were explored using Spearman’s rank correlation analysis where appropriate. Statistical significance was defined as *P < 0.05, **P < 0.01, and ***P < 0.001.

## Results

### OH2 exhibited antitumor efficacy in GBM cells in vitro

**Fig. 1:**
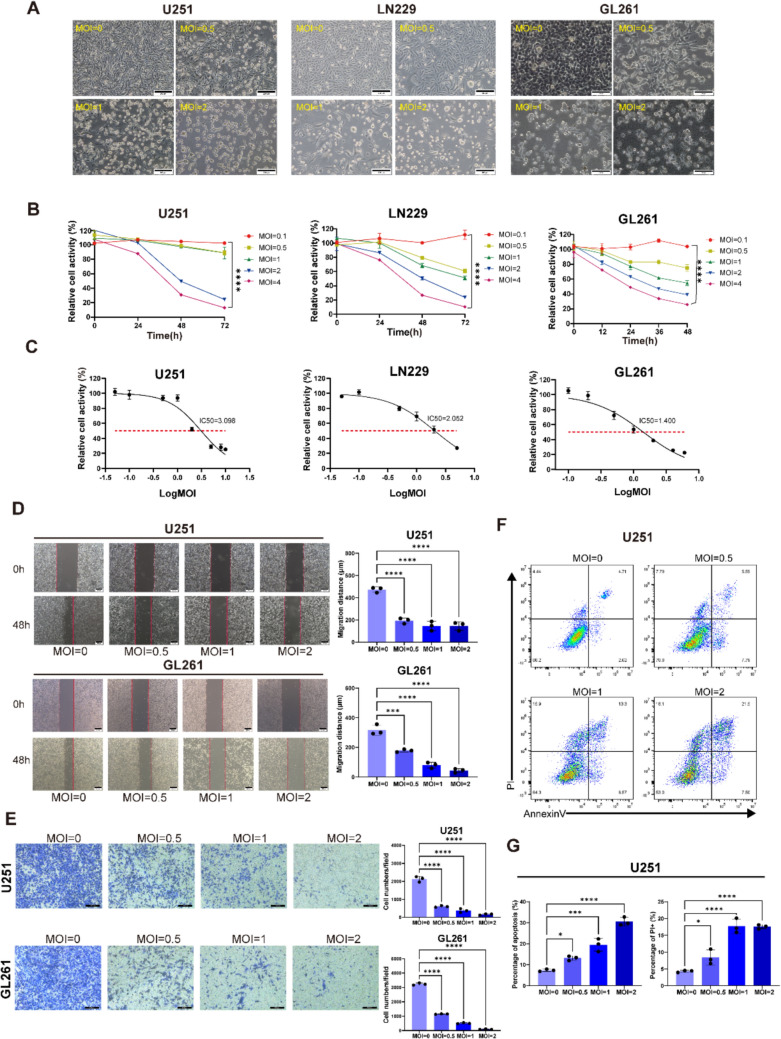
Antitumor effects of OH2 in glioblastoma cells in vitro **A** Morphological alterations of U251, LN229, and GL261 cells following exposure to OH2 at the indicated MOI (MOI = 0, 0.5, 1, 2) for 48h. **B** Cell viability of the three cell lines assessed by the CCK-8 assay. **C** Half-maximal inhibitory concentration (IC_50_) values were determined as MOI = 3.098 (U251), 2.052 (LN229), and 1.400 (GL261). **D**–**E** Wound healing assays and transwell migration assays of U251 and GL261 cells after treatment with OH2 at the indicated MOI (MOI = 0, 0.5, 1, 2) for 48h. Scale bars, 300 μm (wound healing) and 500 μm (transwell migration). **F–G** Flow cytometric analysis of apoptosis in U251 cells exposed to OH2 (MOI = 0, 0.5, 1, 2) for 48h. All experiments were repeated three times. Data shown are from one representative experiment with three parallel samples per group and are presented as mean ± SD. *P < 0.05, **P < 0.01, ***P < 0.001

To evaluate the antitumor effects of OH2, we first examined its impact on GBM cells in vitro. Morphological changes were observed in human (U251 and LN229) and murine (GL261) GBM cell lines following OH2 treatment at increasing MOIs, including cell rounding, enlargement, and loss of protruding spikes (Fig. 1A). CCK-8 assays showed that cell viability in U251, LN229, and GL261 cells decreased in a dose- and time-dependent manner in all three cell lines after OH2 exposure (Fig. 1B). The three GBM cell lines showed different sensitivities to OH2, with IC_50_ values of 3.098, 2.052, and 1.400 MOI at 48h, respectively (Fig. 1C). Based on these results, MOIs of 0.5, 1, and 2 were selected for subsequent in vitro experiments. 3D viability assays also demonstrated that OH2 exposure decreased the proliferation of tumor spheroids (Fig. S1A–B).

To further characterize the antitumor activity of OH2, wound healing assays and Transwell assays were performed to evaluate migratory capacity. The results revealed that OH2 remarkably inhibited the migration of U251 and GL261 cells in a dose-dependent manner (Fig. 1D–E). FCM further demonstrated increased apoptosis in U251 and GL261 cells after 48h of OH2 treatment (Fig. 1F–G, Fig. S1C–D). Notably, an increased proportion of PI-only-positive events was observed, consistent with loss of membrane integrity and oncolytic cell lysis, suggesting that OH2 exerts both apoptotic and lytic cytotoxicity. Cell cycle analysis further indicated that OH2 treatment was associated with S-phase accumulation in U251 cells (Fig. S1E–G).

### OH2 suppressed tumor growth in subcutaneous GBM models and shifted TAMs toward an M1-like phenotype

**Fig. 2:**
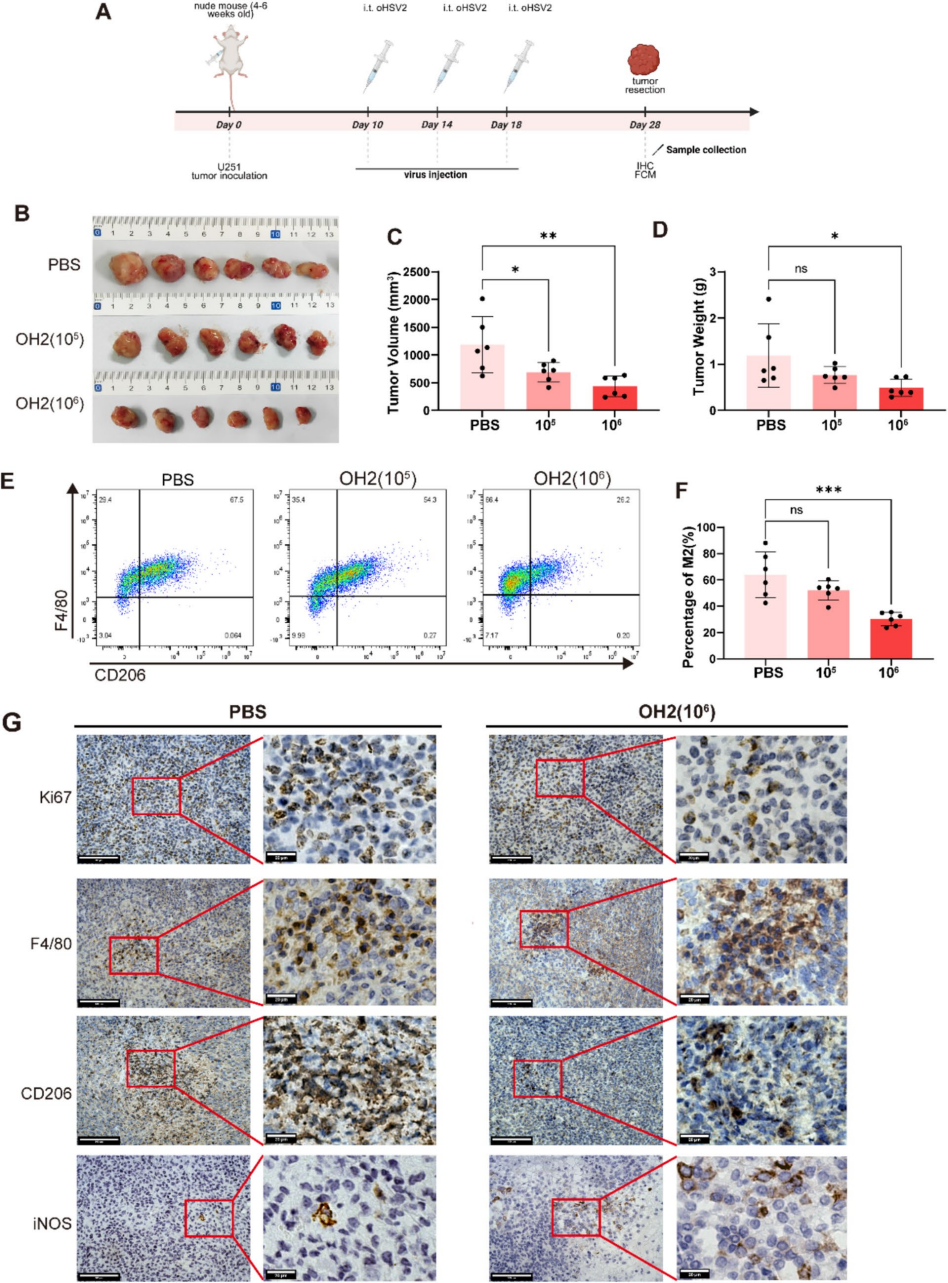
OH2 inhibits tumor progression and promotes M1-like polarization of TAMs in subcutaneous GBM models **A** Experimental timeline and treatment schedule for subcutaneous xenograft models. **B** Images of excised subcutaneous tumors. **C**–**D** Tumor volume and tumor weight in OH2-treated groups (1 × 10^5^ CCID_50_, 1 × 10⁶ CCID_50_) compared with PBS controls. **E** Representative flow cytometry plots of M2-like tumor-associated macrophages (TAMs). **F** Proportion of M2-like TAMs (CD206⁺F4/80⁺) determined by flow cytometry. **G** Representative immunohistochemical (IHC) staining of Ki-67, F4/80, CD206, and iNOS in subcutaneous tumors from OH2 (1 × 10⁶ CCID_50_) and PBS groups. Scale bars, 100 μm (low magnification) and 20 μm (high magnification). Data are presented as mean ± SD. *P < 0.05, **P < 0.01, ***P < 0.001

To assess the in vivo antitumor activity of OH2, we first evaluated tumor growth in subcutaneous GBM models. U251 cells were subcutaneously implanted into the athymic nude mice. Approximately 8 days after inoculation, mice were randomized to receive intratumoral OH2 (1 × 10^5^ CCID_50_ or 1 × 10^6^ CCID_50_) or PBS (Fig. 2A). OH2 significantly reduced tumor volume and tumor weight compared with PBS controls (Fig. 2B–D, Fig. S2A), without significant differences in body weight among groups (Fig. S2B). Histological analysis revealed reduced tumor cell density after OH2 treatment (Fig. S2C), and IHC staining showed a decreased Ki-67 proliferation index in the 1 × 10^6^ CCID_50_ group (Fig. 2G), indicating that OH2 treatment effectively inhibited GBM cell proliferation in vivo.

In parallel, we examined whether OH2 treatment was associated with changes in TAMs. FCM analysis demonstrated that OH2 treatment reduced the percentage of CD206^+^F4/80^+^ cells (M2-like TAMs) compared with PBS controls, while no significant changes were observed in the percentage of CD86^+^F4/80^+^ (M1-like TAMs) (Fig. 2E–F, Fig. S2D). However, the M1/M2 ratio was markedly increased in the 1 × 10^6^ CCID_50_ group (Fig. S2E). Consistently, IHC analysis revealed elevated expression of iNOS (an M1 marker) and reduced expression of CD206 (an M2 marker) in OH2-treated tumors relative to the PBS group (Fig. 2G). These results suggest that OH2 therapy not only inhibits tumor growth but also is associated with a shift toward an M1-like TAM phenotype in subcutaneous GBM models.

### OH2 suppressed tumor growth and regulated TAM phenotype in orthotopic GBM models

**Fig. 3:**
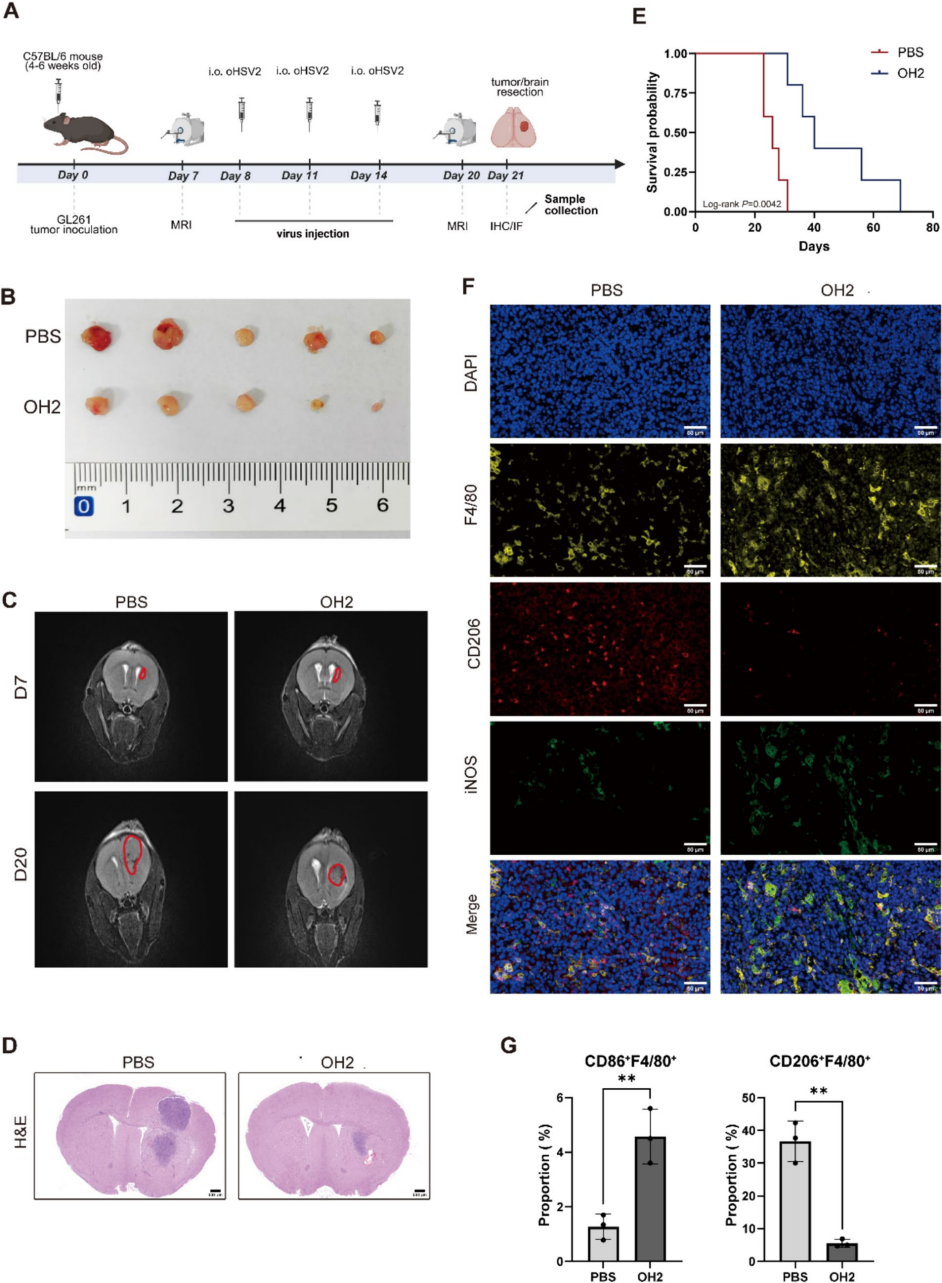
Antitumor effects of OH2 and TAM phenotype modulation in orthotopic GBM models **A** Experimental timeline and treatment schedule for orthotopic GBM models. **B** Representative images of intracranial tumors. **C** Representative images of MRI on days 7 and 20. **D** H&E staining of intracranial tumor sections. Scale bars, 500 μm. **E** Kaplan–Meier survival curves of orthotopic GBM models. **F** Representative multiplex immunofluorescence (mIF) images of intracranial tumor tissues treated with OH2 or PBS. Scale bars, 50 μm. **G** Proportions of CD86^+^F4/80^+^ and CD206^+^F4/80^+^ were quantified. Data are presented as mean ± SD. *P < 0.05, **P < 0.01, ***P < 0.001

To further validate these findings in a more physiologically relevant context, we next evaluated the effects of OH2 in an orthotopic GBM model. GL261 cells were stereotactically implanted into the right striatum of C57BL/6 mice and tumor formation was confirmed by MRI on day 7 after inoculation. OH2 or PBS was administered intratumorally on days 8, 11, and 14, and tumor burden was assessed by MRI on day 20. On day 21, brain and tumor tissues were harvested for FCM, H&E and mIF analyses (Fig. 3A). Representative gross tumor images, MRI, and H&E images suggested reduced tumor burden in OH2-treated mice compared with PBS-treated controls (Fig. 3B–D), consistent with the findings from the subcutaneous tumor models. Kaplan–Meier survival analysis showed that OH2 treatment significantly prolonged the overall survival of tumor-bearing mice in the orthotopic GBM model compared with the control groups (log-rank test, *P* = 0.0042) (Fig. 3E).

mIF results showed increased numbers of F4/80^+^ TAMs in OH2-treated tumors. Notably, OH2 treatment increased iNOS^+^ (M1-like) macrophages and reduced CD206^+^ (M2-like) macrophages within the tumor microenvironment (Fig. 3F–G). These results were consistent with the subcutaneous xenograft model, suggesting enhanced M1-like TAM polarization in vivo. Together, these results demonstrate that OH2 suppresses glioma growth while promoting M1-like TAM polarization in orthotopic GBM models.

### OH2 promoted macrophage proliferation, phagocytosis, and M1-like polarization

**Fig. 4:**
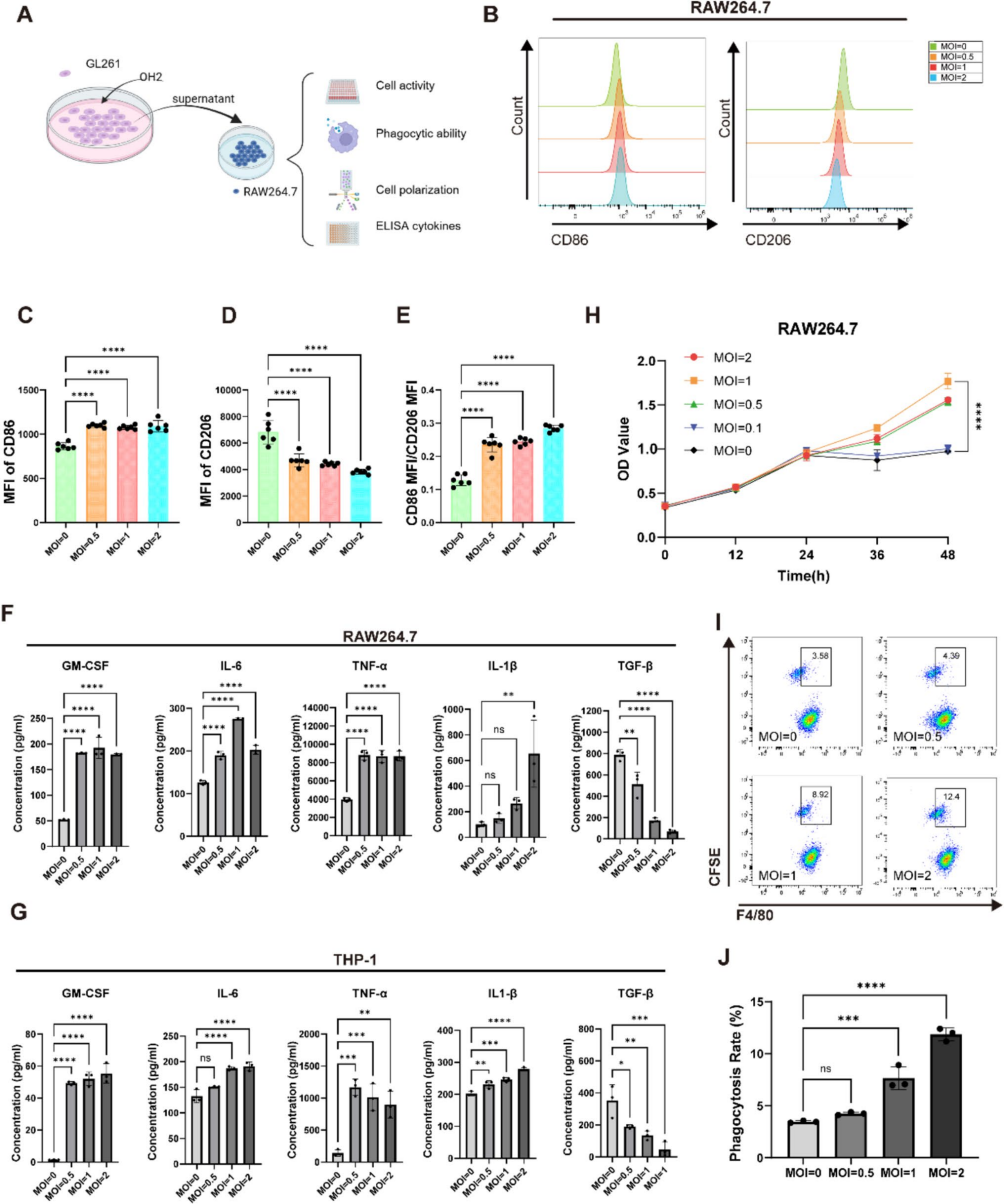
OH2-conditioned medium (CM) induces polarization, promotes proliferation and phagocytic activity of RAW264.7 cells in vitro **A** Schematic workflow of CM preparation using GL261 cells and subsequent in vitro experiments. **B** Representative flow cytometry histograms of mean fluorescence intensity (MFI) of CD86 (M1 marker) and CD206 (M2 marker) in RAW264.7 cells cultured with CM for 48h. **C**–**E** Quantitative analysis of CD86 MFI, CD206 MFI, and the CD86/CD206 MFI ratio. **F**–**G** Cytokine concentrations (GM-CSF, IL-6, TNF-α, IL-1β, TGF-β1) in supernatants of RAW264.7 and THP-1 cells exposed to CM. (H) CCK-8 assay of RAW264.7 proliferation following CM stimulation for 48h. (I-J) Phagocytic capacity of RAW264.7 cells co-cultured with GL261 cells based on CFSE⁺F4/80⁺ percentages. All experiments were repeated three times. Data shown are from one representative experiment and are presented as mean ± SD. *P < 0.05, **P < 0.01, ***P < 0.001

To investigate the mechanisms underlying the TAM-associated changes observed in *vivo*, we examined macrophage responses using CM derived from OH2-treated tumor cells (U251 and GL261) (Fig. 4A, Fig. S2F). FCM analysis revealed that the mean fluorescence intensity (MFI) of CD86 was significantly increased (Fig. 4B–C, Fig. S2G–H), while CD206 expression was decreased (Fig. 4B, D, Fig. S2G, I) in macrophages treated with OH2-CM. In particular, the ratio of MFI of CD86 to CD206 was significantly increased (Fig. 4E). These findings indicate that OH2-CM promoted polarization of macrophages (M0-state) toward the M1-like phenotype. Consistent with these phenotypic changes, ELISA assays showed increased secretion of GM-CSF, IL-6, TNF-α, and IL-1β, and reduced TGF-β1 levels in macrophage cultures treated with OH2-CM (Fig. 4F–G).

In addition, CCK-8 assays demonstrated that RAW264.7 cell proliferation was significantly enhanced after OH2-CM treatment (Fig. 4H), suggesting that OH2 stimulates TAM activation. Phagocytosis assays showed that when RAW264.7 cells pretreated with OH2-CM were co-cultured with CFSE-labeled GL261 cells, the percentage of CFSE^+^F4/80^+^ cells increased in OH2 groups (Fig. 4I–J). These results indicate that OH2-conditioned signals promote macrophage activation, enhance phagocytic capacity, and induce M1-like polarization.

### OH2 promotes M1-like macrophage polarization via JAK-STAT1 signaling

**Fig. 5:**
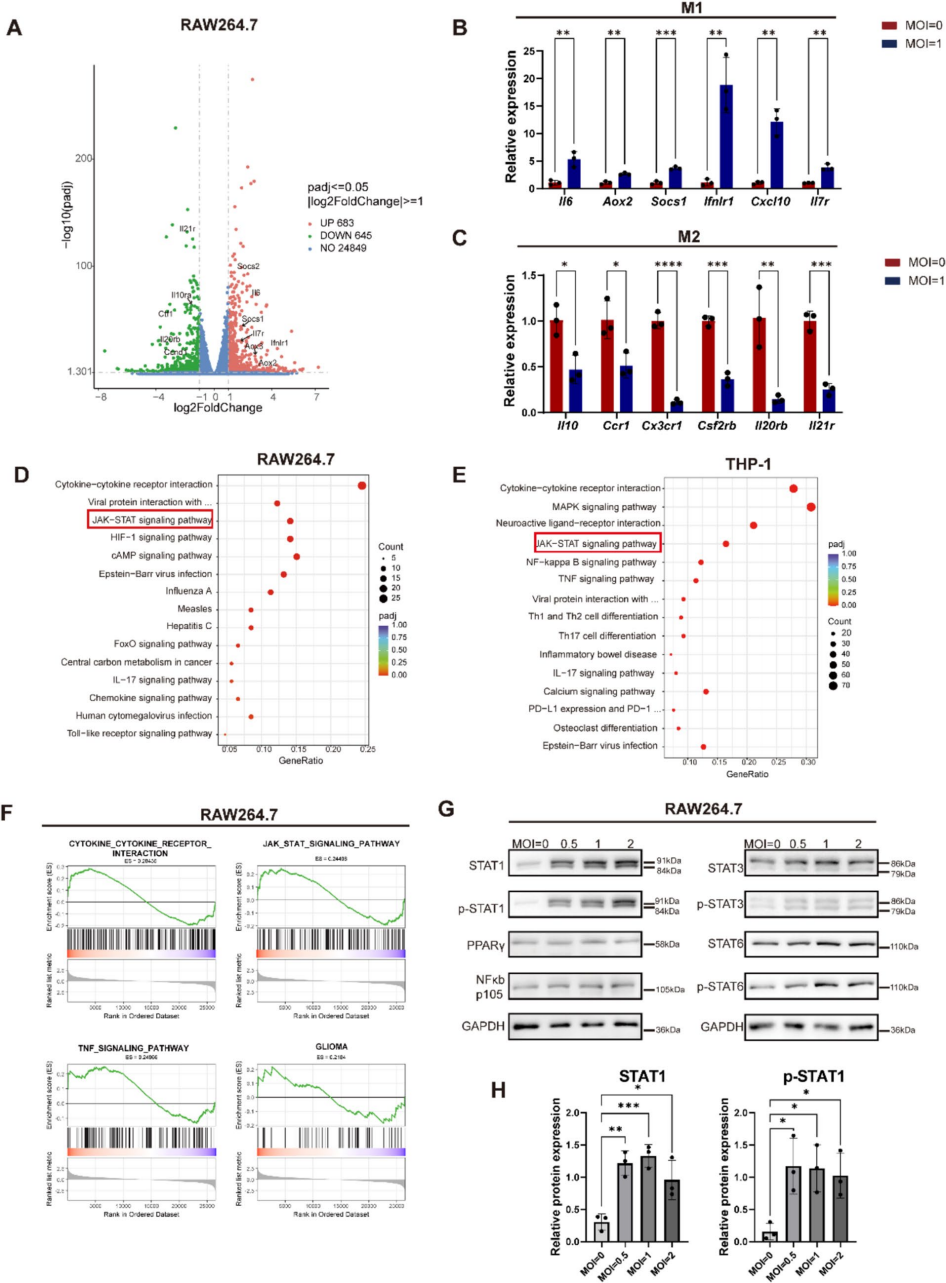
OH2 induces polarization of RAW264.7 cells and modulates the JAK-STAT1 signaling pathway **A** Volcano plot of differentially expressed genes (DEGs) in RAW264.7 cells cultured with CM. **B**–**C** Validation of M1- and M2-related mRNA expression by RT-qPCR in RAW264.7 cells after 48h of CM stimulation. **D**–**E** KEGG enrichment analysis of DEGs in RAW264.7 and THP-1 cells. **F** GSEA analysis of DEGs in RAW264.7 cells. **G** Western blotting analysis of STAT1, p-STAT1, STAT3, p-STAT3, STAT6, p-STAT6, NF-κB, and PPARγ protein levels in RAW264.7 cells. **H** Densitometric quantification of STAT1 and p-STAT1 in RAW264.7 cells. All experiments were repeated three times. Data shown are from one representative experiment. Data are presented as mean ± SD. *P < 0.05, **P < 0.01, ***P < 0.001

To further elucidate the molecular pathways associated with OH2-induced macrophage phenotypic changes, RAW264.7 and THP-1 (M0-state) cells treated with CM (MOI = 0 or 1) underwent transcriptome sequencing. In RAW264.7 cells, 683 genes were upregulated and 645 downregulated, whereas in THP-1 cells, 3,655 genes were upregulated and 3392 downregulated (Fig. 5A, Fig. S3A). In RAW264.7 cells, RT-qPCR validated increased expression of M1-related genes (*Il6, Aox2, Socs1, Ifnlr1, Cxcl10, Il7r*) and decreased expression of M2-related genes (*Il10, Ccr1, Cx3cr1, Csf2rb, Il20rb, Il21r*) (Fig. 5B–C). In THP-1 cells, transcriptomic profiling similarly revealed enrichment of M1-associated gene signatures and suppression of M2-associated signatures (Fig. S3B–C).

GO analysis indicated enrichment of antiviral pathways, including viral response and innate immune regulation (Fig. S3D–E). KEGG and GSEA analyses revealed predominant enrichment of the JAK-STAT pathway, along with TNF, PD-L1 and Th1/Th2 signaling (Fig. 5D–F, Fig. S3F). At the protein level, WB showed marked upregulation of STAT1 and p-STAT1 (Tyr^701^), with no significant changes in STAT3, STAT6, NFκB, or PPARγ (Fig. 5G–H, Fig. S3G–H).

**Fig. 6:**
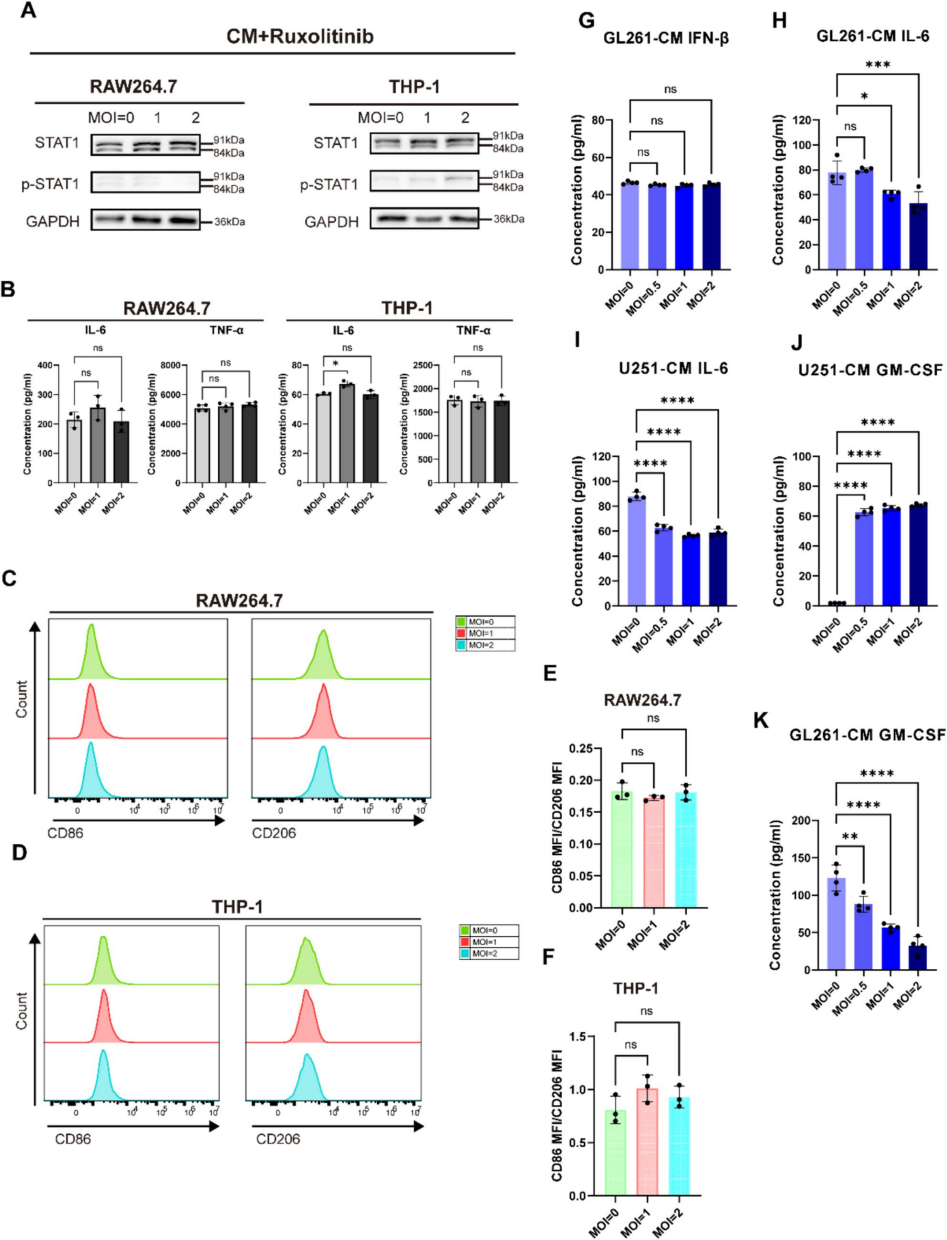
Pharmacologic JAK inhibition attenuates OH2-associated polarization and upstream cytokines exploration of JAK-STAT pathway **A** Western blotting analysis of STAT1 and p-STAT1 protein levels in both RAW264.7 and THP-1 cells treated with CM in the continuous presence of JAK1/2 inhibitor ruxolitinib (5 μM). **B** Concentrations of IL-6 and TNF-α in culture supernatants of RAW264.7 and THP-1 cells exposed to CM in presence of ruxolitinib. **C**–**D** Representative flow cytometry histograms of the MFI of CD86 and CD206 in RAW264.7 and THP-1 cells treated with CM in the presence of ruxolitinib for 48h. **E–F** Quantitative analysis of the CD86/CD206 MFI ratio in RAW264.7 and THP-1 cells under ruxolitinib treatment. **G**–**H** Concentration of IFN-β and IL-6 in GL261-CM. **I**–**J** Concentration of IL-6 and GM-CSF in U251-CM. **K** Concentration of GM-CSF in GL261-CM. All experiments were repeated three times. Data shown are from one representative experiment and are presented as mean ± SD. *P < 0.05, **P < 0.01, ***P < 0.001

To examine the functional contribution of JAK-STAT1 signaling, macrophages were treated with CM in the presence of the JAK1/2 inhibitor ruxolitinib (5 μM). Ruxolitinib markedly suppressed STAT1 phosphorylation, as evidenced by reduced p-STAT1 (Tyr^701^) levels (Fig. 6A). Under JAK inhibition, the differences in IL-6 and TNF-α secretion between OH2-CM and control CM were largely attenuated (Fig. 6B). Consistently, the MFI of CD86 and CD206, as well as the CD86/CD206 ratio, were not significantly altered compared with control groups (Fig. 6C–F). Collectively, these findings suggest that OH2-associated M1-like macrophage polarization is associated with JAK-STAT1 signaling.

We further investigated the classic upstream cytokines associated with the activation of the JAK-STAT1 pathway, including GM-CSF, IFN-α, IFN-β, IFN-γ, and IL-6. The results showed that only low levels of IFN-β were detectable in GL261-CM, with no statistically significant differences between OH2 treatment groups and the control group (Fig. 6G). In U251-CM, none of the three interferons were detected within the detection range of the assay kit. In addition, IL-6 expression in both GL261-CM and U251-CM exhibited a decreasing trend (Fig. 6H–I). Meanwhile, although GM-CSF was significantly elevated in U251-CM upon OH2 treatment (Fig. 6J), it was decreased in GL261-CM (Fig. 6K), suggesting that the increased GM-CSF detected in U251-CM was mostly derived from the human GM-CSF expression sequence inserted in OH2 rather than secretion by GBM cells. Collectively, the classic JAK-STAT1-activating cytokines (GM-CSF, IFN-α/β/γ, and IL-6) did not show an expression pattern consistent with OH2-induced JAK-STAT1 activation, indicating that the upstream factors triggering JAK-STAT1 pathway activation are more likely derived from damage-associated molecular patterns (DAMPs) released by OH2-mediated lysis of GBM cells or other non-classical factors.

### OH2 reshaped the brain immune microenvironment and altered peripheral monocyte profiles

**Fig. 7:**
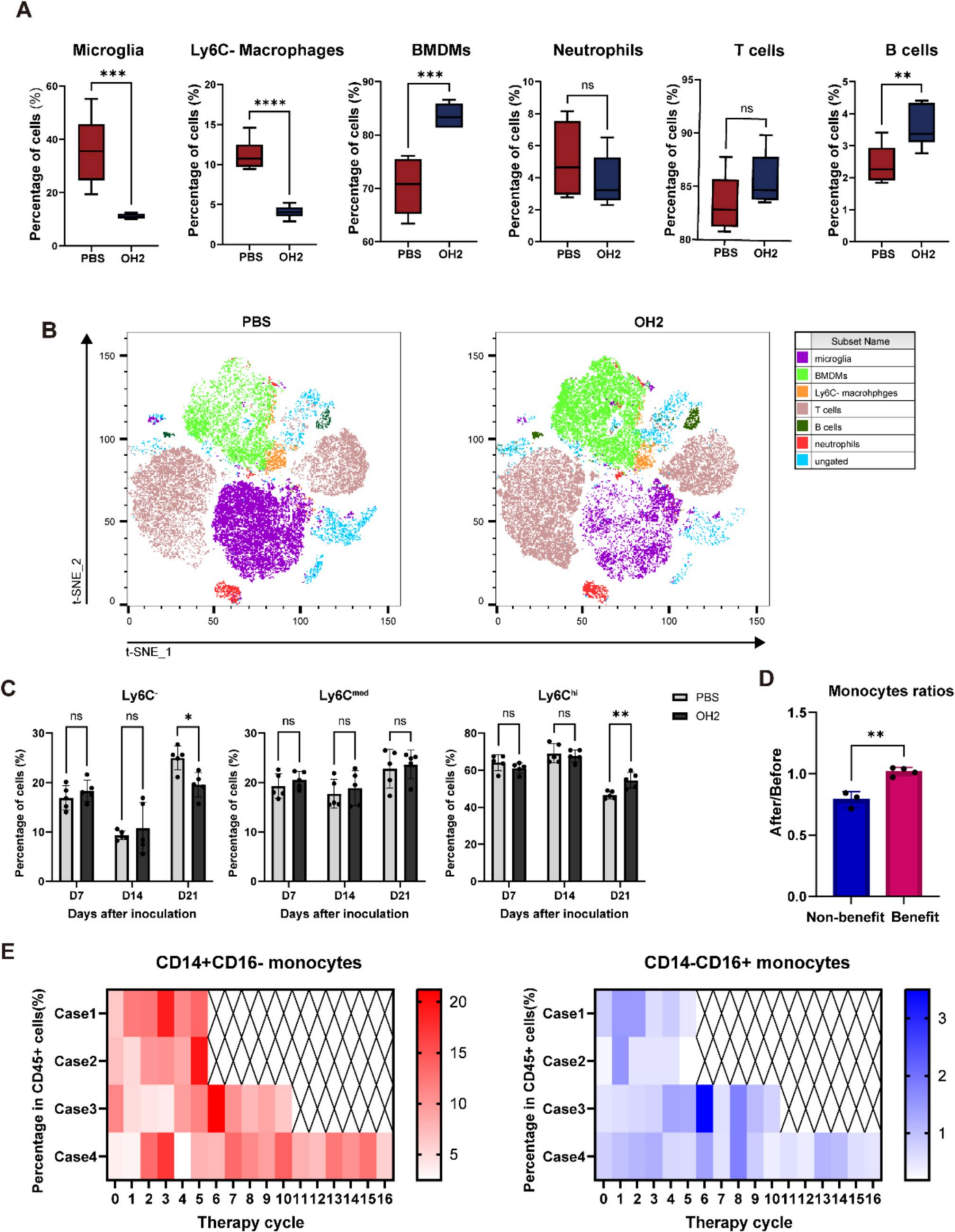
OH2 influences brain immune landscapes and peripheral immunity **A** Flow cytometric quantification of microglia, Ly6C^−^ macrophages, bone marrow-derived macrophages (BMDMs), neutrophils, T cells, and B cells in intracranial xenografts. **B** t-SNE plots of brain-infiltrating immune cells. **C** Proportions of Ly6C⁻, Ly6C^med^, and Ly6C^hi^ monocytes in peripheral blood of mice bearing intracranial tumors. **D** Ratio of post-treatment to pre-treatment monocyte percentage after first cycle of OH2 treatment in 7 patients. **E** Heatmap showing the proportions of peripheral blood monocyte subsets (CD14⁺CD16^−^ and CD14^−^CD16⁺) among clinical benefit group patients across OH2 treatment cycles. Data are presented as mean ± SD. *P < 0.05, **P < 0.01, ***P < 0.001

We next profiled immune cell populations in the tumor-bearing brain following OH2 treatment. On day 21 after inoculation, microglia and brain-infiltrating immune cell subsets were analyzed by multidimensional FCM following tumor removal. Traditional gating (Fig. S4A) revealed a marked decrease in microglia (CD45^med^) and Ly6C^−^ macrophages (F4/80^+^Ly6C^−^), accompanied by significant increases in bone marrow-derived macrophages (BMDMs; F4/80^+^Ly6C^+^) and B cells, whereas neutrophil and T cell proportions were not significantly altered (Fig. 7A). To better visualize these multidimensional data, t-SNE dimensionality reduction and FlowSOM clustering were applied, confirming that OH2 treatment altered the overall immune landscape of the tumor-bearing brain (Fig. 7B).

We further explored the influence of OH2 on peripheral blood monocytes in orthotopic tumor models. FCM was performed on days 7, 14, and 21 to evaluate three monocyte subsets (Ly6C^−^, Ly6C^med^, Ly6C^hi)^, gated as shown in Fig. S4B. Results showed that the proportion of Ly6C^hi^ monocytes was significantly increased, while Ly6C^−^ monocytes were significantly decreased in the OH2 group on day 20 (Fig. 7C). We analyzed the monocyte counts in routine blood tests obtained before Cycle 1 treatment and at the time of response evaluation after Cycle 1 treatment in 7 patients. The results indicated that the post-treatment versus pre-treatment ratio of monocyte percentage appeared to be higher in the clinical benefit group compared with the non-benefit group (Fig. 7D). These findings suggest that monocyte percentage tended to be elevated after treatment in patients of the clinical benefit group, which is consistent with the results observed in peripheral blood from the orthotopic mouse model.

### Exploratory longitudinal immune dynamics and cytokine alterations

We performed exploratory analyses of the longitudinal changes of peripheral blood monocytes in 4 patients of the clinical benefit group. PBMCs were examined across treatment cycles (Fig. S4C). The proportion of CD14⁺CD16^−^ classical monocytes remained essentially stable throughout OH2 treatment cycles (Fig. 7E), suggesting stability of this subset in the observed immune dynamics.

The progression-free survival (PFS) of the 4 patients treated with OH2 was 3.5, 3.5, 7.7, and 12 months, respectively. Using 4 months as the cutoff value, we divided the 4 patients into a long PFS group (≥ 4 months) and a short PFS group (< 4 months). Patients with longer PFS tended to exhibit smaller (flatter) regression slope than those with shorter PFS (Fig. S5A). Exploratory analysis suggested a potential negative association between regression slope and PFS (Spearman’s ρ = − 0.95), suggesting that slower immune trajectories are associated with longer PFS (Fig. S5B).

Exploratory profiling of plasma and CSF cytokines suggested potential associations with PFS. Plasma IL-15, GM-CSF, and IL-10 tended to be higher in patients with longer PFS (Fig. S5C–D), whereas CSF MIP-1β decreased following treatment and appeared lower in longer-PFS cases (Fig. S4E–F). Hierarchical clustering of plasma cytokines suggested a potential separation by PFS (Fig. S4G). Due to the small sample size, correlation analysis was exploratory, but these trends support further evaluation of peripheral immune dynamics and soluble immune mediators as indicators of immune response to OH2 in larger patient cohorts.

## Discussion

GBM remains one of the most challenging malignancies to treat, as conventional therapies have failed to achieve substantial improvements in patient survival [34]. In this study, we systematically demonstrate that the oncolytic virus OH2 exerts antitumor effects in GBM through both direct tumor cell killing and immune modulation, accompanied by M1-like TAM polarization and activation of the JAK-STAT1 signaling pathway. Compared with other OVs such as G47∆ (oHSV-1, approved in Japan) and DNX-2401 (adenovirus, in clinical trials) [35, 36], OH2 represents a distinct oHSV-2 platform with unique immunomodulatory properties, supporting its translational potential in GBM.

In vitro, OH2 induced GBM cell death with both apoptosis and lysis features, consistent with canonical OV properties [37, 38]. Apoptosis processes may promote immune recognition of tumor-associated antigens, while lysis releases tumor-associated antigens (TAAs), together contributing to immunogenic cell death. These dual effects may contribute to coordinated activation of innate and adaptive immunity responses, which are critical for effective virotherapy [38–40]. Consistently, OH2 reduced cell viability and inhibited cell migration, a key feature given that diffuse invasion drives GBM recurrence [41, 42]. However, we acknowledge that glioblastoma cells may undergo multiple forms of cell death, and additional pathways, such as autophagy or cellular stress responses [43], may also contribute and warrant further investigation. In vivo, OH2 suppressed tumor growth in subcutaneous and orthotopic models with favorable safety, in line with prior oHSV studies [11, 13, 44].

Beyond direct oncolysis, OH2 induced a shift of TAMs toward a pro-inflammatory phenotype, characterized by increased CD86, iNOS and pro-inflammatory cytokine expression, along with reduced CD206 and anti-inflammatory cytokines. Transcriptomic and protein analyses indicated activation of the JAK-STAT1 pathway, and pharmacologic JAK inhibition attenuated pro-inflammatory signatures, supporting the involvement of this pathway in this shift, although further studies are required to establish a definitive causal role. Notably, canonical upstream cytokines known to activate JAK-STAT1 did not show a consistent increase in our system. These effects were observed in a CM-based system, suggesting contributions from both tumor-derived and virus-induced factors.

OH2 treatment significantly altered the myeloid composition in the tumor-bearing brain, with reduced immunosuppressive resident microglia and Ly6C^−^ macrophages while increasing infiltration of BMDMs. Notably, Ly6C^hi^ monocytes, widely recognized as inflammatory monocytes with strong migratory capacity[45], were significantly increased in the peripheral blood following OH2 treatment. The temporal association between increased Ly6C^hi^ monocytes in peripheral blood and elevated BMDMs in the brain suggests the possibility that OH2 promotes recruitment of bone marrow-derived inflammatory monocytes to the tumor site. Similarly, patients with clinical benefit maintained elevated levels of CD14⁺CD16⁻ monocytes, suggesting a potentially conserved pattern of monocyte mobilization across species.

Exploratory clinical analyses suggested that longitudinal immune dynamics may have prognostic value. In this small cohort (n = 4), clinical observations are presented descriptively, and no statistical conclusions can be drawn. Nevertheless, patients with relatively stable immune cell dynamics appeared to be associated with prolonged PFS. Plasma cytokine profiling revealed higher levels of IL-15, GM-CSF, and IL-10 in long-PFS patients, whereas CSF MIP-1β showed an inverse association with PFS. Although limited by small sample size, these observations may offer preliminary insights that merit further evaluation in larger cohorts.

Several limitations should be acknowledged. First, the present work is largely based on murine models, which do not fully reflect the complexity of human immunity. Second, the clinical cohort was limited in size, precluding definitive conclusions and highlighting the need for validation in larger patient cohorts. Third, macrophage cell lines do not fully reflect the complexity of primary monocytes or macrophages, and further studies incorporating primary systems will be important to further validate these findings. Finally, pharmacological inhibition does not fully establish the specificity of JAK-STAT1 signaling, and further mechanistic studies are warranted.

## Conclusions

OH2 exhibits antitumor activity in GBM through combined direct oncolytic effects and modulation of the TME. In addition to inhibiting tumor growth, OH2 promotes a shift in TAM phenotypes and is associated with coordinated changes in both brain and peripheral immune compartments. Exploratory clinical observations suggest that longitudinal immune dynamics and cytokine profiles may serve as candidate biomarkers associated with patient outcomes. Although the precise molecular pathways require further investigation, our findings provide a rationale for continued clinical evaluation of OH2 as a therapeutic strategy for GBM.

## Supplementary Information

Below is the link to the electronic supplementary material.

Supplementary file1 (DOCX 3074 KB)

## Data Availability

The datasets generated and analyzed during the current study are available from the corresponding author upon reasonable request.
