# Supplementary material for: A novel oncolytic herpes simplex virus type 2 induces polarization of tumor-associated macrophages and remodels the immune microenvironment in glioblastoma
Source: Cancer Immunol Immunother. 2026 May 30;75(8):200. doi: 10.1007/s00262-026-04440-w (PMC13442803; doi:10.1007/s00262-026-04440-w)
Supplement: Supplementary file 1 [file 262_2026_4440_MOESM1_ESM.docx]

**Supplementary Table 1.** Flow cytometry antibodies.

| **Antibody** | **Manufacturer** | **Cat#** |
| --- | --- | --- |
| AF700 anti-mouse Ly-6G | BioLegend | 127621 |
| APC anti-human CD14 | BioLegend | 301808 |
| APC anti-human CD86 | BioLegend | 374208 |
| APC anti-mouse CD206 | BioLegend | 141708 |
| APC anti-mouse CD3 | BioLegend | 100236 |
| BV421 anti-mouse CD19 | BioLegend | 115549 |
| BV421 anti-mouse CD86 | BioLegend | 105123 |
| FITC anti-human CD45 | BioLegend | 304006 |
| FITC anti-human CD68 | BioLegend | 333806 |
| FITC anti-mouse CD45 | BioLegend | 157608 |
| L/D-FVS780 | BD | 565388 |
| PE anti-human CD16 | BioLegend | 302008 |
| PE anti-human CD206 | BioLegend | 321106 |
| PE anti-mouse CD115 | BioLegend | 135505 |
| PE anti-mouse F4/80 | BioLegend | 157304 |
| PE/Cy7 anti-mouse Ly-6C | BioLegend | 128017 |
| PerCP anti-mouse/human CD11b | BioLegend | 101230 |

**Supplementary Table 2.** WB antibodies.

| **Antibody** | **Manufacturer** | **Cat#** |
| --- | --- | --- |
| STAT1 | CST | 14994S |
| p-STAT1-tyr701 | CST | 9167S |
| STAT3 | CST | 9139S |
| p-STAT3-tyr705 | CST | 9145S |
| STAT6 | CST | 5397S |
| p-STAT6-tyr641 | CST | 56554S |
| NF-κB p50/p105 | Proteintech | 14220-1-AP |
| PPARγ | Proteintech | 16443-1-AP |
| GAPDH | Proteintech | 60004-1-Ig |
| β-actin | Proteintech | 66009-1-Ig |
| HRP-linked anti-rabbit IgG | CST | 7074S |
| HRP-linked anti-mouse IgG | CST | 7076S |

**Supplementary Table 3**. Primers used for qPCR.

| **Gene** | **Forward** **(5'→3')** | **Reverse** **(5'→3')** |
| --- | --- | --- |
| *Aox2* | CATTGTTGATGCGGTGGCTG | GGTCATCTCTCGGGATCATGT |
| *Ccr1* | CACTCACCGTACCTGTAGCC | GCAAATATCAGACGCACGGC |
| *Csf2rb* | CATGACCCTCCTCTATCACCAG | GAGATACACTCCATTCCAGCAG |
| *Cx3cr1* | ACGCTGACTCCTCATTCCAC | CTCCCCAATGTGCAAGCAAC |
| *Cxcl10* | AAGCTATGTGGAGGTGCGAC | AACCCCTTGGGAAGATGGTG |
| *Ifnlr1* | GTCTGGGAAGCAAGACCCTG | GTATAGACGGTTCTGGCGCT |
| *Il10r* | AGGATGTTGTCGCGTTTGC | GGGCTTGGCAGTTCGTATG |
| *Il20rb* | TTAACCGAAATGCAACTGTCCTC | CCTCCAGTAGACCACAAGGAAC |
| *IL21r* | CTTATGACGAACCCTCCAACTAC | CTCACAGCATAGGGGTCTCTG |
| *Il6* | AGTCCTTCCTACCCCAATTTCC | TGGTCTTGGTCCTTAGCCAC |
| *Il7r* | ACCCAAGAATCAAGGAGGATGG | GGGAGACTAGGCCATACGAC |
| *Socs1* | CTCCTTGGGGTCTGTTGGC | GCGTGCTACCATCCTACTCG |
| *Gapdh* | AGGTCGGTGTGAACGGATTTG | TGTAGACCATGTAGTTGAGGTCA |

**Supplementary Table 4.** IHC and mIF antibodies.

| **Antibody** | **Manufacturer** | **Cat#** | **Dilution rate** |
| --- | --- | --- | --- |
| Ki67 (IHC) | Proteintech | 28074-1-AP | 1:1000 |
| iNOS (IHC) | CST | 68186 | 1:100 |
| CD206 (IHC/mIF) | Proteintech | 18704-1-AP | 1:800 |
| F4/80 (IHC) | Proteintech | 28463-1-AP | 1:2500 |
| F4/80 (mIF) | Abcam | AB300421 | 1:50 |
| iNOS (mIF) | CST | 68186 | 1:200 |
| HRP-labeled goat anti-rabbit IgG | Servicebio | G1215 | 1:50 |

**Fig. S1 Effects of OH2 on proliferation of 3D tumor spheroids, apoptosis in GL261 cells and cell cycle progression in U251 cells**

(A-B) Viability assessed by luminescence signal in tumor spheroids of GL261 and U251 cells using CellTiter-Glo 3D Cell Viability Kit. Scale bars, 500 μm. (C-D) Flow cytometric analysis of apoptosis in GL261 cells treated with increasing concentrations of OH2 (MOI = 0, 0.5, 1, 2) for 48 h. (E-G) Flow cytometric analysis of cell cycle distribution in U251 cells following OH2 treatment (MOI = 0, 0.5, 1, 2) for 48 h. All experiments were repeated three times. Data shown are from one representative experiment with three parallel samples per group and are presented as mean ± SD. **P* < 0.05, ***P* < 0.01, ****P* < 0.001.

**Fig. S2 OH2 suppresses tumor progression in subcutaneous GBM models and induces TAM polarization**

(A) Tumor volume measurements every three days starting from day 6 post U251 cells inoculation in subcutaneous xenograft models. (B) Body weight monitoring of mice every three days throughout the experiment. (C) Representative H&E staining of subcutaneous tumor sections from OH2-treated groups (1×10⁵ CCID50, 1×10⁶ CCID50) and PBS controls. Scale bars, 50 μm (D) Percentage of M1-like TAMs within subcutaneous tumor tissues. (E) Ratio of M1/M2 TAMs in subcutaneous tumors. (F) Schematic illustration of CM preparation using U251 cells and the experimental workflow for *in vitro* studies. (G) Representative flow cytometry histograms of CD86 and CD206 MFI in THP-1 cells cultured with CM for 48 h. (H-I) Quantitative analysis of CD86 and CD206 MFI. All experiments were repeated three times. Data shown are from one representative experiment and are presented as mean ± SD. **P* < 0.05, ***P* < 0.01, ****P* < 0.001.

**Fig. S3 OH2-CM modulates transcriptional responses and activates JAK-STAT signaling pathway**

(A) Volcano plot showing DEGs in THP-1 cells exposed to CM. (B-C) Expression of M1- and M2-associated marker in THP-1 cells following CM stimulation. (D-E) GO enrichment analysis of DEGs in RAW264.7 and THP-1 cells treated with CM. (F) KEGG pathway enrichment analysis of DEGs in THP-1 cells treated with CM. (G) Western blot analysis of STAT1, p-STAT1, STAT3, STAT6, NF-κB, and PPARγ protein expression in THP-1 cells treated with CM. (H) Densitometric quantification of STAT1 and p-STAT1 in THP-1 cells. All experiments were repeated three times. Data shown are from one representative experiment with three parallel samples per group and are presented as mean ± SD. **P* < 0.05, ***P* < 0.01, ****P* < 0.001.

**Fig. S4 Flow cytometry gating strategies**

(A) Representative gating strategy for brain-infiltrating immune cells in mice. (B) Representative gating strategy for peripheral blood monocytes in mice. (C) Representative gating strategy for peripheral blood monocytes in humans.

**Fig. S5 Peripheral immune dynamics and cytokine profiling in plasma and CSF show potential associations with patient outcome**

(A) Individual patient trajectories of peripheral CD14⁺CD16⁻ monocyte proportions over time with linear regression fits and corresponding PFS. (B) Correlation between regression slope and PFS. (C) Volcano plot of plasma cytokines identifying IL-15, GM-CSF, and IL-10 as differentially expressed cytokines. (D) Correlation between plasma cytokine levels (GM-CSF, IL-10, IL-15) and PFS. (E) Volcano plot of CSF cytokines identifying MIP-1β as differentially expressed cytokines. (F) Correlation between CSF cytokine MIP-1β and PFS. (G) Heatmap illustrating longitudinal expression patterns of significant plasma cytokines in plasma (row z-score).

**Fig. S1 Effects of OH2 on proliferation of 3D tumor spheroids, apoptosis in GL261 cells and cell cycle progression in U251 cells**

**Fig. S2 OH2 suppresses tumor progression in subcutaneous GBM models and induces TAM polarization**

**Fig. S3 OH2-CM modulates transcriptional responses and activates JAK-STAT signaling pathway**

**Fig. S4 Flow cytometry gating strategies**

**Fig. S5 Peripheral immune dynamics and cytokine profiling in plasma and CSF show potential associations with patient outcome**
